# The Need for Education

**Published:** 1920-07-24

**Authors:** Ronald C. Macfie


### THE NEED FOR EDUCATION.

*To the Editor of* The Hospital.

Sir,—In your issue of July 17 a correspondent deplores "that so many men must, of sheer necessity, accept life-long employment in occupations which entail chronic constipation (look at the pills sold), excessive physical strain, or practically no exercise at all, a cutting off from sunshine and fresh air, or, worse still, rotting in some form or other from chemicals." There is cause to deplore, but does not your correspondent rather exaggerate? Undoubtedly, there are unhealthy occupations which "debar men from varied and balanced mental and physical activity," and may even lead to disease and premature death; but these are few, and the mental and physical degeneration so common in industrial cities is due not so much to occupation as to causes within the control of the industrial worker or industrial community. Take consumption, that disease so common in industrial cities. That dire disease is a disease not so much of occupation as of unhygienic habits and homes. It attacks the mother in the home as much as the father in the factory, and at present it is raging in Jugo-Slavia, which is not an industrial nation at all; but simply a nation ignorant of hygiene. Take, again, poor physique. It is true that most industrial workers are puny, but that again is mainly due to unsuitable food and insufficient exercise and insufficient fresh air, and these causes are rather economic and educational than occupational.

A few occupations entail excessive physical strain; but is there any which deprives the worker of sunshine and fresh air and precludes all exercise? I doubt it. It is perfectly true that many factories and workshops have insufficient light and fresh air, but that is the fault of the factory or workshop, not of the particular occupation, and, in any case, no occupation prevents an open bedroom window, and eight hours of fresh air by night will make up for a good deal of foul air by day. As to lack of exercise. There are, of course, sedentary occupations—such as, tailoring or journalism—but even the tailor may run to his shop and the journalist may sprint to his office.

Most of the wrecks one finds in all industrial cities have been wrecked, not by the hardships of their work but by the conditions under which they have worked and lived in their factories and homes, and by the diseases and vices that cities breed. When they have been broken down by the work itself, that has been due not so much to the nature of the work as to the fatigue consequent on long hours, and salvation will be found in shorter hours and avoidance of fatigue by a regulation of work on physiological principles. In future most workers will have short hours (unless in times of war), and will have ample time for bodily and mental recreation, and it is not impossible that in future the hours of play will be more perilous than the hours of labour. Industries and industrial specialisation cannot be abolished without danger to the commonwealth and common health; but every effort should be made to give the industrial worker healthy conditions of work and to teach him how to employ his leisure to profit both his body and his soul. Especially in the case of those whose work is purely mechanical is it necessary to encourage various artistic and intellectual interests.

I sympathise with much of your correspondent's letter, but, as I have said, I think he rather exaggerates, and I think that what is required is not a reformation of the industries, nor garden cities, so much as the hygienic education of the industrial workers and a practical application both in factory and home of hygienic principles.

Yours faithfully,

Ronald C. Macfie, M.A., M.B.

